# Personalized therapy in multiple sclerosis: an Italian Delphi consensus

**DOI:** 10.1007/s00415-025-13173-2

**Published:** 2025-05-27

**Authors:** Claudio Gasperini, Diego Centonze, Antonella Conte, Paolo Gallo, Alessandra Lugaresi, Francesco Patti, Maria Trojano, Maria Pia Amato, Massimo Filippi

**Affiliations:** 1 Department of Neurosciences, S Camillo Forlanini Hospital, Rome, Italy; 2 Department of Systems Medicine, Tor Vergata University, Rome, Italy; 3 IRCCS Neuromed, Pozzilli, Isernia Italy; 4 Department of Human Neurosciences, Sapienza, University of Rome, Rome, Italy; 5 Department of Neuroscience, University of Padova, Padua, Italy; 6 Azienda Ospedaliera of Padua, Padua, Italy; 7 Department of Biomedical and Neuromotor Sciences, Università di Bologna, Bologna, Italy; 8 IRCCS Istituto delle Scienze Neurologiche di Bologna, Bologna, Italy; 9 Department of Medical and Surgical Sciences and Advanced Technologies, GF Ingrassia, University of Catania, Catania, Italy; 10 Azienda Ospedaliero-Universitaria Policlinico “G. Rodolico-S. Marco”, Catania, Italy; 11 Department of Translational Biomedicine and Neurosciences-DiBraiN, University of Bari “Aldo Moro”, Bari, Italy; 12 Department NEUROFARBA, University of Florence, Florence, Italy; 13 IRCCS Fondazione Don Carlo Gnocchi, Florence, Italy; 14 Neuroimaging Research Unit, Division of Neuroscience, IRCCS San Raffaele Scientific Institute, Milan, Italy; 15 Neurology Unit, IRCCS San Raffaele Scientific Institute, Via Olgettina, 60, 20132 Milan, Italy; 16 Neurorehabilitation Unit, IRCCS San Raffaele Scientific Institute, Milan, Italy; 17 Neurophysiology Service, IRCCS San Raffaele Scientific Institute, Milan, Italy; 18 Vita-Salute San Raffaele University, Milan, Italy

**Keywords:** Multiple sclerosis, Delphi, Personalized treatment

## Abstract

**Objective:**

The increasing availability of disease-modifying therapies (DMTs) may provide more personalized treatment options for multiple sclerosis (MS) based on various factors, including patients’ characteristics, prognostic indicators, comorbidities, and safety. In Italy, recent efforts focused on promoting interdisciplinary, patient-centered care and equitable access to optimized therapies, as reported in the *2023 Barometer of Multiple Sclerosis and Related Diseases* from the Italian Multiple Sclerosis Association. A key challenge is ensuring equitable access to homogeneous and personalized therapeutic strategies.

**Materials and methods:**

Using a Delphi methodology, a panel of Italian neurologists with expertise in MS evaluated consensus on specific aspects of MS treatments, including personalized therapy, patient involvement in decision-making, treatment flexibility, self-management of therapies, perception of treatment efficacy and safety and therapeutic sequence management.

**Results:**

Of 166 votes, 116 statements reached consensus (68% positive, 2% negative), representing 70% of the total, whereas 50 (30%) highlighted areas of non-consensus. The findings emphasize the central role of neurologists, the importance of personalized therapy, the inclusion of patients in therapeutic choices to enhance adherence and quality of life, and managing both quality of life and caregiver burden. Most high-efficacy disease-modifying therapies (HE DMTs), like cladribine and anti-CD20 therapies, recognized for their efficacy and convenience of administration, received positive consensus, emphasizing their perceived value in individualized treatment approaches.

**Conclusions:**

This research highlights best practices and provides a roadmap for improving patient outcomes through tailored, well-communicated therapeutic strategies.

## Introduction

Multiple sclerosis (MS) is a chronic, progressive neurological disorder affecting the central nervous system (CNS), characterized by heterogeneous clinical manifestations, disease course and accumulation of irreversible clinical disability [1]. Pathologically, MS is defined by inflammation, demyelination, and neurodegenerative processes that can begin during the early stages, often subclinically and underrecognized [1].

Several disease-modifying therapies (DMTs) are currently available to slow the accumulation of irreversible disability [2–8]. In recent years, treatment landscape and strategies for MS have substantially evolved with the introduction of increasingly effective DMTs [2–8], each with distinct mechanisms of action, allowing for personalized treatment strategies tailored to the unique characteristics of individual patients.

The choice of DMT is guided by several factors, including patient demographics, clinical presentation and features, prognostic factors (clinical, biological, and radiological), comorbidities, as well as patient preferences and lifestyle. Additionally, treatment decisions are shaped by existing clinical guidelines [4, 5], availability of DMTs (often limited by reimbursement policies and regulatory approvals), and safety considerations.

In recent years, considerable efforts have been made in Italy to enhance interdisciplinary, patient-centered care, promote full social inclusion, and advance MS research and communication. These initiatives are aimed at empowering both healthcare professionals involved in MS management and patients affected by this disease. The *2023 Barometer of Multiple Sclerosis and Related Diseases*, a comprehensive report from the Italian Multiple Sclerosis Association (AISM), provides a detailed snapshot of MS in Italy, highlighting also unmet patient needs that should be addressed in the next few years [9]. Among the key challenges identified there is the necessity to ensure equitable access to optimized, personalized therapeutic strategies that should be homogeneous across the whole Country. To address this unmet need, it is necessary to establish a strong consensus regarding the consideration of MS as a severe disease having profound impact on the quality of life of not only MS patients, but also their families and caregivers. This consensus serves as a foundation for promoting equitable, homogeneous access to personalized therapeutic strategies throughout Italy. Moreover, in response to these challenges, the present study seeks to address the treatment needs of MS patients, as identified by AISM, and to reach a consensus on different statements based on these patient-driven insights. Moreover, the study aims to evaluate the alignment between individual patient needs and the different DMT options available, with the ultimate goal of optimizing personalized treatment. Finally, the study aims to gauge expert consensus on patient-centric therapeutic decision-making in MS care using a Consensus Delphi methodology. Even though the study focuses on the Italian healthcare context, the core principles may inform international approaches with appropriate adaptation.

## Methods

### Study design and Delphi methodology

This study aimed to gauge the level of consensus on a set of predefined statements using the Delphi methodology, following guidance from published Delphi reporting guidelines [10, 11]. The Delphi technique is a structured communication method designed to achieve consensus or assess the degree of agreement among experts by using iterative rounds of questionnaires [12, 13].

An Italian panel of Key Opinion Leaders (KOLs), acknowledged as the authors of this paper and recognized as leading experts in MS field, developed the questionnaire (Box 1).

**Box 1:** Statements of the Delphi questionnaire

**Part 1: Introduction**
**Statement #1: Regarding the value of personalized therapy in MS, I believe that:**
1.1 The neurologist’s role in evaluating therapies with the MS patient is central, both in terms of effectiveness and side effects. All MS patients must receive a therapy consistent with their clinical condition, which also takes into account their expectations and life needs
1.2 Personalizing treatment can optimize effectiveness and reduce side effects, improving compliance, adherence, persistence, and quality of life
1.3 Personalized therapy management should take into account the specific disabilities and needs of the person with MS
**Statement #2: Regarding inclusion and participation in therapeutic choices, I believe these are priority goals:**
2.1 Providing adequate information through effective communication
2.2 Involving the patient transparently in therapeutic choices to improve quality of life
2.3 Promoting information on parenthood at the time of MS diagnosis
2.4 Estimating and discussing the prognosis at the time of diagnosis to improve therapeutic decisions
2.5 Making the patient an active participant in therapeutic choices to enhance long-term treatment adherence
2.6 Valuing adherence to treatments to maximize therapeutic effects and to maintain quality of life for people with MS
**Part 2: Patient Needs: Personalized Approaches**
**Statement #3: Regarding flexibility (travel, work, family planning, managing comorbidities), I believe that:**
3.1 Given that MS is often diagnosed in young adults, the disease impacts life plans, such as family planning, work, and lifestyle
3.2 Regarding flexibility (travel, work, family planning, managing comorbidities), I believe that treatment with:
– Alemtuzumab is an optimal choice
– Cladribine is an optimal choice
– Dimethyl fumarate is an optimal choice
– Fingolimod is an optimal choice
– Glatiramer acetate is an optimal choice
– Interferon is an optimal choice
– Natalizumab is an optimal choice
– Ocrelizumab is an optimal choice
– Ofatumumab is an optimal choice
– Ozanimod is an optimal choice
– Ponesimod is an optimal choice
– Siponimod is an optimal choice
– Teriflunomide is an optimal choice
3.3 Women of reproductive age with MS may find it difficult to reconcile their desire for parenthood with their condition and treatment choices. For this statement, I believe that treatment with:
– Alemtuzumab is an optimal choice
– Cladribine is an optimal choice
– Dimethyl fumarate is an optimal choice
– Fingolimod is an optimal choice
– Glatiramer acetate is an optimal choice
– Interferon is an optimal choice
– Natalizumab is an optimal choice
– Ocrelizumab is an optimal choice
– Ofatumumab is an optimal choice
– Ozanimod is an optimal choice
– Ponesimod is an optimal choice
– Siponimod is an optimal choice
– Teriflunomide is an optimal choice
3.4 The Treatment choice should take into account the patient’s short-term desire for pregnancy, when this is compatible with disease activity and prognosis. In this sense, I believe that treatment with:
– Alemtuzumab is an optimal choice
– Cladribine is an optimal choice
– Dimethyl fumarate is an optimal choice
– Fingolimod is an optimal choice
– Glatiramer acetate is an optimal choice
– Interferon is an optimal choice
– Natalizumab is an optimal choice
– Ocrelizumab is an optimal choice
– Ofatumumab is an optimal choice
– Ozanimod is an optimal choice
– Ponesimod is an optimal choice
– Siponimod is an optimal choice
– Teriflunomide is an optimal choice
3.5 Therapeutic choice can also influence quality of life through improved relationship satisfaction
3.6 For a patient with comorbidities, I believe that treatment with:
– Alemtuzumab is an optimal choice
– Cladribine is an optimal choice
– Dimethyl fumarate is an optimal choice
– Fingolimod is an optimal choice
– Glatiramer acetate is an optimal choice
– Interferon is an optimal choice
– Natalizumab is an optimal choice
– Ocrelizumab is an optimal choice
– Ofatumumab is an optimal choice
– Ozanimod is an optimal choice
– Ponesimod is an optimal choice
– Siponimod is an optimal choice
– Teriflunomide is an optimal choice
**Statement #4: Regarding self-management of therapy (home setting vs hospital therapy), I believe that:**
4.1 Self-care by the patients can lead to improved quality of life
4.2 Most patients receiving home therapy report a positive impact on quality of life through self-management of care. In this regard, I believe that treatment with:
– Cladribine is an optimal choice
– Dimethyl fumarate is an optimal choice
– Fingolimod is an optimal choice
– Glatiramer acetate is an optimal choice
– Interferon is an optimal choice
– Ofatumumab is an optimal choice
– Ozanimod is an optimal choice
– Ponesimod is an optimal choice
– Siponimod is an optimal choice
– Teriflunomide is an optimal choice
4.3 Most patients who receive treatment at the Center value the opportunity to talk with doctors and nurses and to share experiences with other patients. In this sense, I believe that treatment with:
– Alemtuzumab is optimal
– Natalizumab is optimal
– Ocrelizumab is optimal
4.4 Some patients find logistical difficulties and challenges in physically attending the Center. For these patients treatment with:
– Alemtuzumab is an optimal choice
– Cladribine is an optimal choice
– Dimethyl fumarate is an optimal choice
– Fingolimod is an optimal choice
– Glatiramer acetate is an optimal choice
– Interferon is an optimal choice
– Natalizumab is an optimal choice
– Ocrelizumab is an optimal choice
– Ofatumumab is an optimal choice
– Ozanimod is an optimal choice
– Ponesimod is an optimal choice
– Siponimod is an optimal choice
– Teriflunomide is an optimal choice
**Part 3: Patient Needs: Quality of Life**
**Statement #5: Regarding caregiver burden, I believe that:**
5.1 The unpredictable progression of MS generates stress in family life
5.2 As the individual’s disability progression increases, the caregiver’s role often becomes more burdensome
5.3 Caregivers play a crucial role in patient management by providing daily assistance, thus the quality of life of caregivers is important
5.4 Caregiver burden is strongly influenced by depressive symptoms, disability level, and limitations in the patient’s physical quality of life
5.5 Improving the patient’s psychosocial conditions positively impacts the quality of life of caregivers
**Statement #6: Regarding quality of life, I believe that:**
6.1 To promote a better quality of life, MS care should include management of fatigue and cognitive disorders. To this end, I believe that treatment with:
– Alemtuzumab is an optimal choice
– Cladribine is an optimal choice
– Dimethyl fumarate is an optimal choice
– Fingolimod is an optimal choice
– Glatiramer acetate is an optimal choice
– Interferon is an optimal choice
– Natalizumab is an optimal choice
– Ocrelizumab is an optimal choice
– Ofatumumab is an optimal choice
– Ozanimod is an optimal choice
– Ponesimod is an optimal choice
– Siponimod is an optimal choice
– Teriflunomide is an optimal choice
6.2 Effective therapies for fatigue and cognitive disorders can reduce indirect costs related to increased patient self-sufficiency
**Part 4: Patients’ Needs: Therapy**
**Statement #7: Concerning the perception of treatments’ efficacy and safety, I believe that:**
7.1 To improve treatment outcomes, it is fundamental that patients perceive the therapies they receive as safe and effective
7.2 It is essential to clearly communicate the risks and benefits of the treatment
7.3 The neurologist must place utmost importance on adverse events, both in the short and long term, in communication with the patient. In this sense, I believe that:
– This is particularly true with regards to alemtuzumab
– This is particularly true with regards to cladribine
– This is particularly true with regards to dimethyl fumarate
– This is particularly true with regards to fingolimod
– This is particularly true with regards to glatiramer acetate
– This is particularly true with regards to interferon
– This is particularly true with regards to natalizumab
– This is particularly true with regards to ocrelizumab
– This is particularly true with regards to ofatumumab
– This is particularly true with regards to ozanimod
– This is particularly true with regards to ponesimod
– This is particularly true with regards to siponimod
– This is particularly true with regards to teriflunomide
7.4 An effective and safe treatment involves fewer therapeutic switches. In this sense, I believe that:
– This is particularly true with regards to alemtuzumab
– This is particularly true with regards to cladribine
– This is particularly true with regards to dimethyl fumarate
– This is particularly true with regards to fingolimod
– This is particularly true with regards to glatiramer acetate
– This is particularly true with regards to interferon
– This is particularly true with regards to natalizumab
– This is particularly true with regards to ocrelizumab
– This is particularly true with regards to ofatumumab
– This is particularly true with regards to ozanimod
– This is particularly true with regards to ponesimod
– This is particularly true with regards to siponimod
– This is particularly true with regards to teriflunomide
7.5 The efficacy in reducing relapses and the tolerability of treatments improve adherence to therapy. In this sense, I believe that:
– This is particularly true with regards to alemtuzumab
– This is particularly true with regards to cladribine
– This is particularly true with regards to dimethyl fumarate
– This is particularly true with regards to fingolimod
– This is particularly true with regards to glatiramer acetate
– This is particularly true with regards to interferon
– This is particularly true with regards to natalizumab
– This is particularly true with regards to ocrelizumab
– This is particularly true with regards to ofatumumab
– This is particularly true with regards to ozanimod
– This is particularly true with regards to ponesimod
– This is particularly true with regards to siponimod
– This is particularly true with regards to teriflunomide
**Statement #8: In the choice of treatment for MS, both the current clinical condition and possible future therapeutic options should be considered:**
8.1 Concerning the future management of therapeutic sequences, I believe that treatment with:
– Alemtuzumab is an optimal choice
– Cladribine is an optimal choice
– Dimethyl fumarate is an optimal choice
– Fingolimod is an optimal choice
– Glatiramer acetate is an optimal choice
– Interferon is an optimal choice
– Natalizumab is an optimal choice
– Ocrelizumab is an optimal choice
– Ofatumumab is an optimal choice
– Ozanimod is an optimal choice
– Ponesimod is an optimal choice
– Siponimod is an optimal choice
– Teriflunomide is an optimal choice
8.2 The correct strategy for therapeutic sequences must take into account the control of clinical/radiological relapses
8.3 The correct strategy for therapeutic sequences must consider AEs/poor tolerability
8.4 The correct strategy for therapeutic sequences must take into account family planning
8.5 The correct strategy for therapeutic sequences must consider personal/work-related needs

*AE* adverse event, *MS* multiple sclerosis

The panel included nine Italian KOLs with recognized expertise in MS, selected based on their scientific output, clinical leadership roles, and geographical distribution across Italy. Most participants are affiliated with major academic centers and specialized MS clinics, collectively managing approximately 50% of MS patients nationally [14]. While community and private practice neurologists were not represented, the selected panel reflects current national leadership in MS care. All participants disclosed potential conflicts of interest (see ‘Conflicts of interest’ section), which were transparently managed through independent data handling and blinded voting procedures.

The 35 items included in the questionnaire were developed through iterative expert discussions during a preparatory meeting and were directly informed by priority areas outlined in the “*2023 Barometer of Multiple Sclerosis and Related Diseases*” [9] from the AISM. The items reflect both literature-based gaps and real-world unmet needs identified by patients and advocacy groups.

Agreement levels were measured using a 5-point Likert scale [15–19]: 1 (“strongly disagree”), 2 (“disagree”), 3 (“slightly agree”), 4 (“agree”), and 5 (“strongly agree”). The consensus threshold was set at 77%, corresponding to 7 out of 9 votes, in line with thresholds commonly used in Delphi literature for small expert panels [20]. This cut-off was chosen to balance the need for strong agreement while allowing for some individual variability. Agreement was considered reached when the combined scores for items 3, 4, and 5 exceeded 77%. Conversely, disagreement was determined when the combined scores for items 1 and 2 surpassed 77%. If neither the agreement (items 3, 4, and 5) nor the disagreement (items 1 and 2) reached 77%, no consensus was identified.

This study employed a modified Delphi approach, characterized by a single round of anonymous voting followed by a structured group discussion during a consensus meeting. Although traditional Delphi methods recommend multiple rounds, we intentionally adopted a streamlined design to enhance efficiency and reduce participant fatigue. Importantly, the post-voting meeting enabled the clarification of borderline items and facilitated reflection on areas of divergence, thus partially compensating for the absence of additional voting rounds.

### Consensus evaluation

Ultimately, eight statements articulated through 35 items were identified, distributed across four areas of interest: Introduction: 2 statements (9 items); Patient Needs: Personalized Approaches: 2 statements (10 items); Patient Needs: Quality of Life: 2 statements (6 items); Patient Needs: Therapy: 2 statements (10 items).

For some statements, a grading evaluation on the available molecules was required. The grading pertained to individual molecules used in current MS treatments: moderate-efficacy (ME) DMTs (glatiramer acetate, interferons, dimethyl fumarate, and teriflunomide) and high-efficacy (HE) DMTs (alemtuzumab, cladribine, fingolimod, natalizumab, ocrelizumab, ofatumumab, ozanimod, ponesimod, and siponimod).

The Delphi questionnaire was administered in English, and the KOLs voted anonymously and independently on each item. The Delphi process consisted of a single voting round, with results discussed in a subsequent meeting.

## Results

### Personalized therapy

**Fig. 1:**
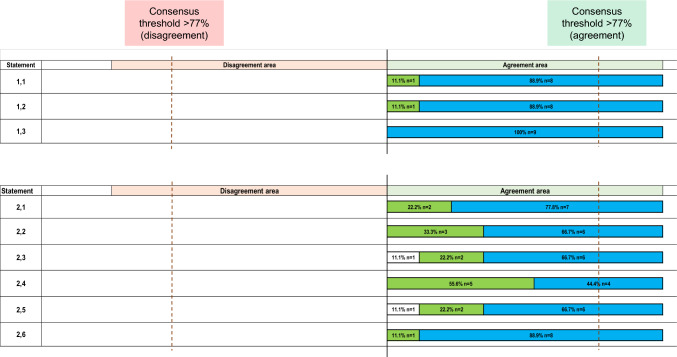
Agreement or disagreement rate (%) by Likert scale score of the expert panel on each statement for Part 1—Introduction of the Delphi questionnaire. Strongly disagree is shown in black, disagree in light gray with dots, slightly agree in white, agree in green, strongly agree in light blue. See text for further details

The central role of neurologists in evaluating therapies, including the assessment of efficacy, tolerability and safety, was unanimously endorsed (100%) (Statement 1.1) (Fig. 1). Likewise, the need for personalized treatment approaches to optimize therapeutic effectiveness, minimize side effects, and improve patient compliance, adherence, persistence, and overall quality of life also received full consensus (100%) (Statement 1.2) (Fig. 1). Additionally, the unanimous agreement (100%) reaffirmed the importance of tailoring therapy based on the specific disabilities and needs of MS patients (Statement 1.3) (Fig. 1).

### Inclusion and participation in therapeutic choices

Unanimous agreement (100%) supported the importance of providing adequate information through effective communication (Statement 2.1) (Fig. 1). Similarly, full consensus (100%) was achieved on the necessity of transparently involving patients in therapeutic decision-making to improve their quality of life (Statement 2.2) (Fig. 1). Furthermore, full agreement (100%) supported the need to provide information regarding parenthood at MS diagnosis (Statement 2.3) (Fig. 1), to estimate and discuss prognosis early to guide therapeutic choices (Statement 2.4) (Fig. 1), and to actively engage MS patients in therapeutic decisions to foster long-term treatment adherence (Statement 2.5) (Fig. 1). Furthermore, the recognition of adherence as crucial to maximizing treatment outcomes and maintaining quality of life in MS patients was also fully endorsed (100%) (Statement 2.6) (Fig. 1).

### Flexibility in treatment use

**Fig. 2:**
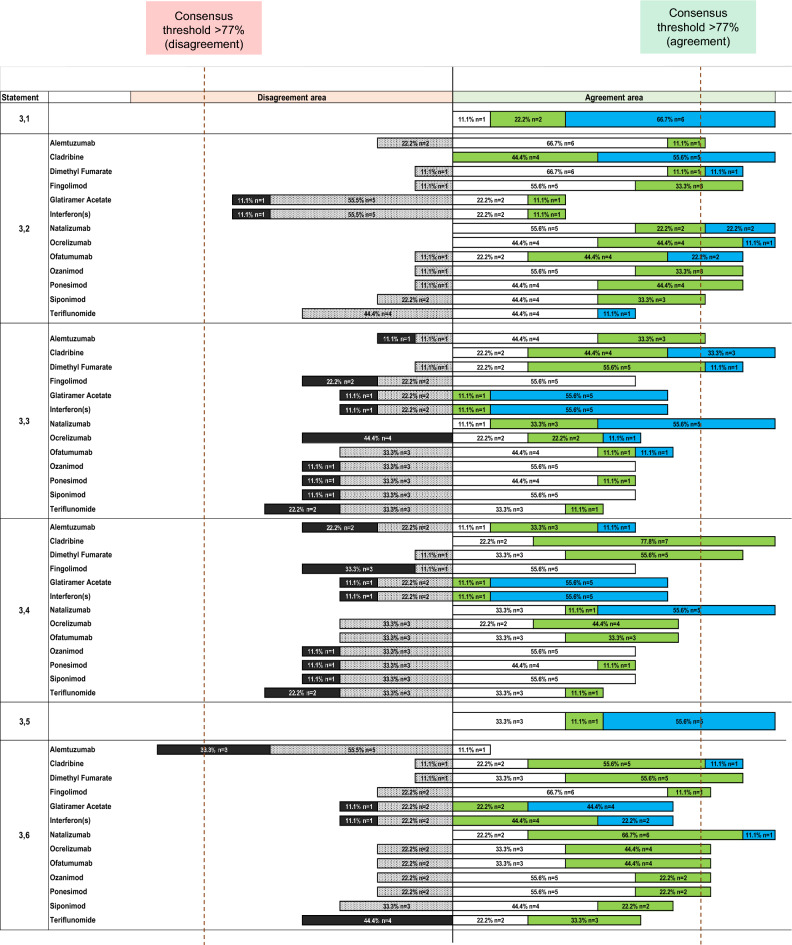
Agreement or disagreement rate (%) by Likert scale score of the expert panel on statement 3 for Part 2—Patient Needs: Personalized Approaches of the Delphi questionnaire. Strongly disagree is shown in black, disagree in light gray with dots, slightly agree in white, agree in green, strongly agree in light blue. See text for further details

There was unanimous agreement (100%) that MS significantly impacts major life plans, including family planning, work, and lifestyle (Statement 3.1) (Fig. 2).

Regarding treatment flexibility, full positive consensus (100%) was achieved for cladribine, natalizumab, and ocrelizumab (Statement 3.2) (Fig. 2). Positive consensus was also observed for ofatumumab (89%), fingolimod (89%), ozanimod (89%), ponesimod (89%), dimethyl fumarate (89%), siponimod (78%), and alemtuzumab (78%). No consensus was reached for glatiramer acetate and interferons (67% disagreement), or for teriflunomide (56% agreement).

For women of reproductive age with MS, full positive consensus (100%) was achieved for cladribine and natalizumab (Statement 3.3) (Fig. 2). Positive consensus was achieved for dimethyl fumarate (89%), and alemtuzumab (78%). No consensus was reached for ofatumumab (67% agreement), glatiramer acetate (67% agreement), interferons (67% agreement), ocrelizumab (56% agreement), fingolimod (56% agreement), ozanimod (56% agreement), ponesimod (56% agreement), siponimod (56% agreement), or teriflunomide (56% disagreement).

Full positive consensus (100%) supported the impact of therapeutic choice on quality of life, particularly through improving relationship satisfaction (Statement 3.5) (Fig. 2).

In MS patients with comorbidities, full positive consensus (100%) was achieved for natalizumab (Statement 3.6) (Fig. 2). Positive consensus was also reached for cladribine (89%), dimethyl fumarate (89%), ocrelizumab (78%), ofatumumab (78%), fingolimod (78%), ozanimod (78%), and ponesimod (78%). Negative consensus was reached for alemtuzumab, with 89% disagreement. No consensus was obtained for siponimod (67% agreement), glatiramer acetate (67% agreement), interferons (67% agreement), or teriflunomide (56% agreement).

### Self-management of therapy

**Fig. 3:**
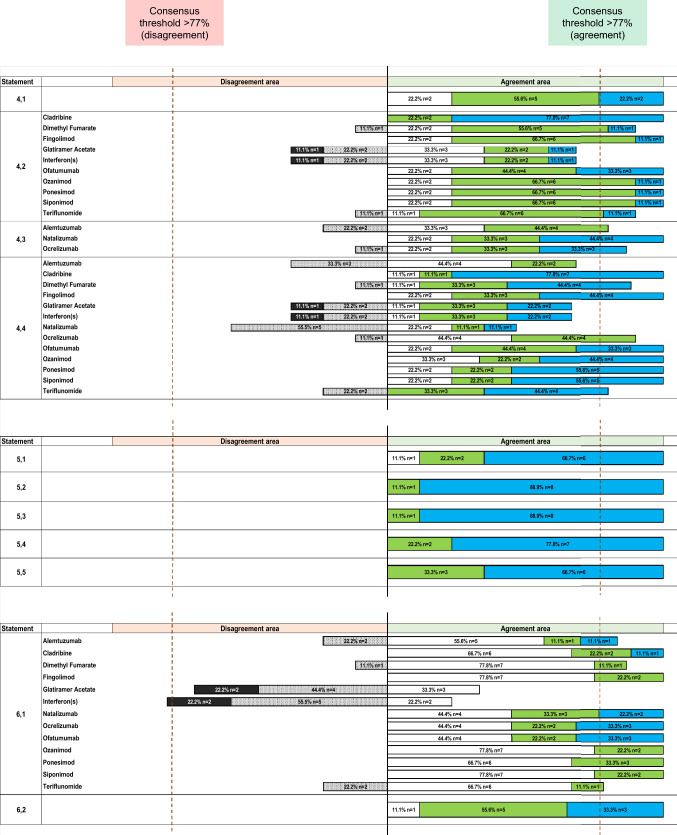
Agreement or disagreement rate (%) by Likert scale score of the expert panel on statement 4 for Part 2—Patient Needs: Personalized Approaches and on statements 5 and 6 for Part 3—Patient Needs: Quality of Life of the Delphi questionnaire. Strongly disagree is shown in black, disagree in light gray with dots, slightly agree in white, agree in green, strongly agree in light blue. See text for further details

The concept that patient self-care improves quality of life received unanimous agreement (100%) (Statement 4.1) (Fig. 3).

Regarding the positive impact of home therapy, full positive consensus (100%) was achieved for cladribine, fingolimod, ofatumumab, ozanimod, ponesimod, and siponimod (Statement 4.2) (Fig. 3). Positive consensus was also reached for dimethyl fumarate (89%) and teriflunomide (89%). No agreement was reached for glatiramer acetate and interferons (67% agreement).

Most patients valued center-based interactions with healthcare providers and with other patients. For this, full positive consensus (100%) was achieved for natalizumab (Statement 4.3) (Fig. 3). Positive consensus was also observed for ocrelizumab (89%) and alemtuzumab (78%).

For patients facing logistical challenges in attending the center, full positive consensus (100%) was achieved for cladribine, fingolimod, ofatumumab, ozanimod, ponesimod, and siponimod as optimal treatment choices (Statement 4.4) (Fig. 3). Positive consensus was also achieved for ocrelizumab (89%), dimethyl fumarate (89%), and teriflunomide (78%). No agreement was reached for alemtuzumab (67% agreement), glatiramer acetate (67% agreement), or natalizumab (56% disagreement).

Additionally, full positive consensus (100%) recognized that the unpredictable course of MS induces family stress (Statement 5.1) (Fig. 3), and that caregiver burden escalates with disability progression (Statement 5.2) (Fig. 3). The critical role of caregivers in daily assistance and the acknowledgment of their quality of life were also fully endorsed (Statement 5.3) (Fig. 3). Caregiver burden related to depressive symptoms, the patient’s level of disability, and limitations in the patient’s physical quality of life similarly achieved unanimous agreement (100%) (Statement 5.4) (Fig. 3).

To promote a better quality of life, MS care must address fatigue and cognitive disorders. Regarding therapeutic choices, full positive consensus (100%) was achieved for cladribine, fingolimod, natalizumab, ocrelizumab, ofatumumab, ozanimod, ponesimod, and siponimod as optimal choices (Statement 6.1) (Fig. 3). Positive consensus was also reached for dimethyl fumarate (89%), alemtuzumab (78%), and teriflunomide (78%). Negative consensus was reached for interferons (78% disagreement). No agreement was reached for glatiramer acetate (67% disagreement).

Effective therapies reducing fatigue and cognitive disorders were unanimously acknowledged as crucial to lowering indirect costs by increasing patient independence (Statement 6.2) (Fig. 3).

### Perception of treatment efficacy and safety

**Fig. 4:**
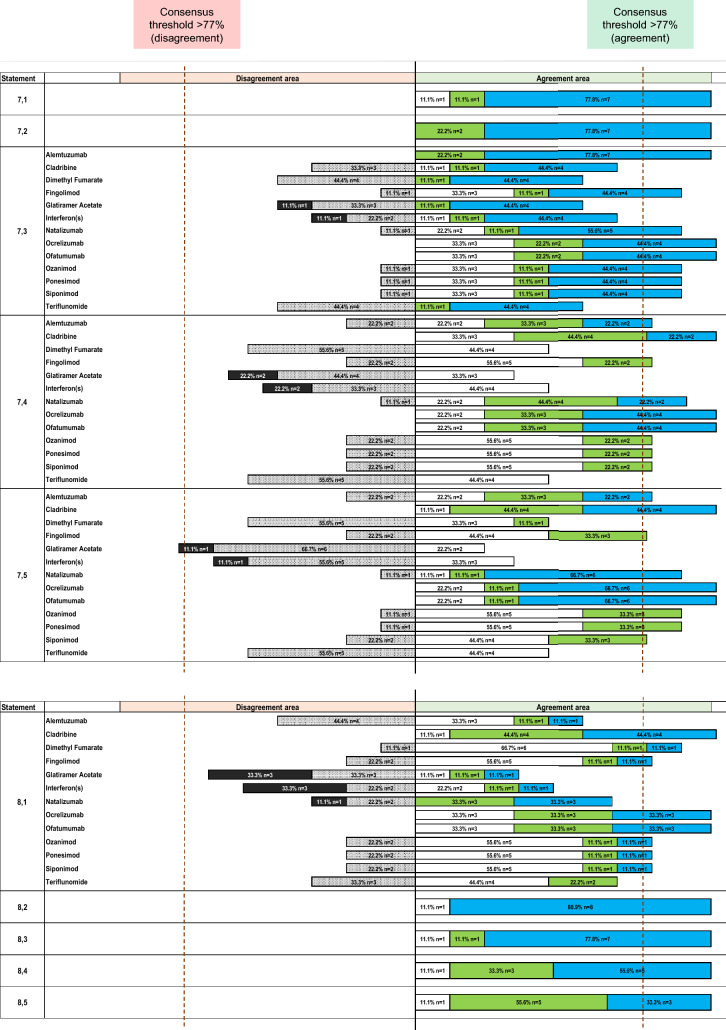
Agreement or disagreement rate (%) by Likert scale score of the expert panel on statement 7 for Part 4—Patients’ Needs: Therapy of the Delphi questionnaire and on statement 8 for Part 4—Patients’ Needs: Therapy. Strongly disagree is shown in black, disagree in light gray with dots, slightly agree in white, agree in green, strongly agree in light blue. See text for further details

The necessity for patients to perceive their therapies as safe and effective received unanimous agreement (100%) (Statement 7.1) (Fig. 4), alongside the importance of clearly communicating treatment risks and benefits (Statement 7.2) (Fig. 4).

Concerning the neurologist’s focus on adverse events when communicating with patients, full consensus (100%) was achieved for alemtuzumab, ocrelizumab, and ofatumumab (Statement 7.3) (Fig. 4). Positive consensus was also reached for natalizumab (89%), fingolimod (89%), ozanimod (89%), ponesimod (89%), and siponimod (89%). No consensus was reached for cladribine (67% agreement), dimethyl fumarate (56% agreement), glatiramer acetate (56% agreement), interferons (67% agreement), and teriflunomide (56% agreement).

Regarding the relationship between effective, safe treatments and fewer therapeutic switches, full positive consensus (100%) for cladribine, ocrelizumab, and ofatumumab (Statement 7.4) (Fig. 4). Positive consensus was also reached for natalizumab (89%), alemtuzumab (78%), fingolimod, ozanimod, ponesimod, and siponimod (78%). No agreement was reached for glatiramer acetate (67% disagreement), dimethyl fumarate (56% disagreement), interferons (56% disagreement), or teriflunomide (56% disagreement).

Reducing relapses and enhancing tolerability to improve treatment adherence also received full positive consensus (100%) for cladribine, ocrelizumab, and ofatumumab as optimal treatment options (Statement 7.5) (Fig. 4). Positive consensus was also reached for natalizumab (89%), ozanimod (89%), ponesimod (89%), alemtuzumab (78%), fingolimod (78%), and siponimod (78%). No consensus was reached for interferons (67% disagreement), dimethyl fumarate (56% disagreement), and teriflunomide (56% disagreement). Negative consensus was reached for glatiramer acetate (78% disagreement).

### Therapeutic sequence management

Full positive consensus (100%) was achieved for cladribine, ocrelizumab, and ofatumumab in future therapeutic sequencing (Statement 8.1) (Fig. 4). Positive consensus was also reached for dimethyl fumarate (89%), fingolimod (78%), and ozanimod (78%), ponesimod (78%), and siponimod (78%). No agreement was reached for glatiramer acetate (67% disagreement), alemtuzumab (56% agreement), interferons (56% disagreement), natalizumab (67% agreement), or teriflunomide (67% agreement).

Additionally, therapeutic strategies were unanimously agreed (100%) to require control over clinical and radiological relapses (Statement 8.2) (Fig. 4), and careful consideration of adverse events and tolerability (Statement 8.3) (Fig. 4).

Family planning (Statement 8.4) and addressing personal and work-related needs (Statement 8.5) were also fully endorsed in planning therapeutic sequences (100%) (Fig. 4).

## Discussion

Out of 166 votes of this Delphi analysis, 116 statements reached consensus (68% positive, 2% negative), representing 70% of the total, whereas 30% highlighted areas of non-consensus. Overall, the Delphi voting results demonstrate strong alignment among healthcare professionals on the importance of personalized therapy, patient involvement, and managing both quality of life and caregiver burden. Most HE DMTs received positive consensus, emphasizing their perceived value in individualized treatment approaches.

### Personalized therapy

There was unanimous agreement that neurologists should play a central role in evaluating MS therapies, particularly regarding efficacy and side effects [21]. This also emphasizes their importance in tailoring treatments to optimize outcomes and minimize adverse effects [22, 23]. The consensus on personalized treatment reflects the need to adapt therapies based on individual patient characteristics, disability levels, and personal needs [22, 23]. Personalized approaches improve not only efficacy but also treatment adherence [23, 24], quality of life, and long-term health, ensuring that therapy addresses both disease management and the MS patient’s unique context [22, 23].

### Inclusion and participation in therapeutic choices

Unanimous agreement on the importance of patient inclusion in decision-making further emphasizes the value of shared decision-making in MS care [25]. Clear communication about prognosis, parenthood, and treatment options enhances patient participation, which improves adherence and satisfaction [24, 25].

Effective communication fosters better understanding and long-term adherence to treatment plans, further strengthening the neurologist’s role as both an educator and clinician [21, 24, 25].

### Flexibility in treatment use

The consensus on MS treatments reflects the evolving landscape of therapeutic options [1, 26, 27]. Full positive consensus on treatment flexibility (Statement 3.2) was achieved for cladribine, natalizumab, and ocrelizumab, highlighting confidence in their flexibility and effectiveness. However, therapies like glatiramer acetate, interferons, and teriflunomide lacked consensus, reflecting ongoing debate about their use, especially in cases requiring flexibility or reproductive planning. For instance, slow elimination of teriflunomide was noted as limiting its flexibility, even though the accelerated elimination procedure could overcome this limitation. Moreover, despite its strong safety profile and absence of major systemic adverse effects, glatiramer acetate did not achieve positive consensus as a flexible option for patients with comorbidities. This likely reflects perceptions of limited efficacy in complex cases, where HE DMTs are preferred to slow disability progression and enhance quality of life. Additionally, the need for frequent injections may reduce its acceptability among patients with higher treatment burdens. Some experts interpreted "family planning" as pregnancy, while others included unexpected pregnancy. Comorbidity factors may not have been fully considered [28, 29], though another section addresses this. The result could also be explained in terms of efficacy: more effective therapies, by promoting a greater sense of well-being for the patient, allow for greater flexibility.

For women of reproductive age (Statement 3.3), cladribine, used as indicated in the Summary of Product Characteristics (SmPC), and natalizumab achieved unanimous consensus, with natalizumab’s evidence supported by clinical practice and published data [30, 31]. Among ME DMTs, only dimethyl fumarate reached positive consensus, despite not being specified in the SmPC, but consistent with clinical practice and neurologists’ discretion, based on the risk–benefit ratio. Interferons did not reach consensus due to side effects impacting quality of life, despite approval for use during pregnancy and breastfeeding. Separate consideration of pregnancy and breastfeeding might have changed the outcome. The perception of drug efficacy and convenience likely influenced these results. Ocrelizumab and ofatumumab did not reach consensus as optimal treatment choices for women of reproductive age with MS. Even though this seems in contrast with recent evidence [32–34], it should be noted that at the time of voting, data on the safety of using ocrelizumab and ofatumumab in women of childbearing age who were seeking pregnancy during treatment were still limited. This uncertainty likely contributed to the lack of consensus on their suitability for this population.

Regarding treatment selection for patients with comorbidities (Statement 3.6), all HE DMTs, except for siponimod, received positive consensus. Lack of consensus of siponimod, compared to ozanimod and ponesimod, may be influenced by the fact that patients treated with siponimod tend to be older, with a longer disease duration, greater disability, and a higher risk of comorbidities. Dimethyl fumarate was the only ME DMT with positive consensus, while alemtuzumab had negative consensus, likely due to safety concerns [1, 35].

These variances suggest some DMTs are less favored in specific contexts, especially regarding reproductive health and comorbidities [28, 29]. The full agreement on therapies such as cladribine for patients with reproductive considerations highlights the need for flexible treatment plans tailored to a patient’s life stage and plans.

### Self-management of therapy

The consensus that patient self-care improves quality of life underscores the importance of empowering patients in chronic disease management. Full positive consensus for therapies like cladribine, fingolimod, ofatumumab, ozanimod, ponesimod, and siponimod (Statement 4.2) reflects a preference for oral or easily administered treatments, reducing the logistical burden on MS patients.

Encouraging self-management enhances autonomy and improves adherence by making treatments more convenient and compatible with MS patients’ lives [24].

In Statement 4.3, natalizumab achieved full consensus, with alemtuzumab (78%) and ocrelizumab (89%) also showing positive consensus. The preference for natalizumab among patients valuing healthcare provider interaction highlights the need for diverse treatment options to accommodate varying patient preferences.

For patients facing logistical challenges in accessing treatment centers (Statement 4.4), consensus was reached for ME and HE oral therapies and HE injectables. Consensus was not reached for ME injectable therapies, alemtuzumab, and natalizumab, possibly due to the multi-day administration regimen of alemtuzumab or biases about treatment efficacy.

Unanimous recognition of the negative impact of MS disability accrual on family life and caregivers reflects the need to consider caregiver well-being in treatment planning. As MS progresses, caregiver support becomes central, highlighting the importance of therapies that reduce disability progression and support patient independence to alleviate caregiver burden.

To improve quality of life, MS care should focus on managing fatigue and cognitive disorders (Statement 6.1). Full consensus was achieved for all HE DMTs, except alemtuzumab that received positive consensus, which was also achieved for oral ME DMTs. Interferons received negative consensus, whereas glatiramer acetate failed to reach consensus. These results are due to side effects of specific treatments like interferons on fatigue and on literature supporting their more limited effects on preserving cognitive functions.

### Perception of treatment efficacy and safety

While reducing relapses and improving tolerability are general goals applicable across treatments, the experts’ perception reflected differences in the extent to which each DMT achieves these goals. High-efficacy and better-tolerated treatments were viewed as more likely to foster long-term adherence. The unanimous agreement on the importance of patients perceiving their therapies as safe and effective highlights the psychological aspect of treatment adherence [24]. Confidence in the ability of the treatment to manage symptoms without causing harm increases long-term persistence. Effective communication about risks and benefits is crucial, emphasizing the neurologist’s role in building trust and understanding.

Neurologists must prioritize clear communication to help patients feel confident in their treatment, as this positively impacts adherence [24]. Treatments with fewer side effects and causing less adverse events are more likely to result in long-term adherence, minimizing the need for frequent therapeutic switches and improving overall MS management [24].

Regarding the focus on side effects and adverse in patient communication (Statement 7.3), none of the ME DMTs achieved consensus. The only HE DMT that did not reach consensus was cladribine. Since KOLs fully agreed that this treatment is generally well tolerated and relatively safe, the lack of agreement likely stems from the heterogeneous interpretation of the question. Given its good safety profile, there is no need for further discussion regarding side effects, as concerns over tolerability and safety are limited.

For the statement that effective, safe treatments lead to fewer therapeutic switches (Statement 7.4), cladribine and anti-CD20 therapies were the only ones to achieve 100% consensus. HE DMTs generally received positive consensus, reflecting broad agreement on their effectiveness and suitability in MS management.

Reducing relapses and improving tolerability are key to improve adherence (Statement 7.5). Fewer relapses contribute to a more stable disease course, improving patient quality of life. Better tolerability and limited risk of side effects may foster consistent use, higher effectiveness, and higher adherence rates [24]. All HE DMTs received positive consensus, with cladribine and anti-CD20 therapies achieving 100% consensus. In contrast, none of the ME DMTs reached positive consensus. Accordingly, HE DMTs favor long-term adherence [24].

### Therapeutic sequence management

In managing MS treatment sequences (Statement 8.1), cladribine and anti-CD20 therapies achieved 100% consensus, with most HE DMTs, except for alemtuzumab and natalizumab, receiving positive consensus. Among ME DMTs, only dimethyl fumarate obtained positive consensus, while the others did not. The result for interferons and glatiramer acetate were somewhat surprising in the context of sequencing, as these therapies typically allow for easier transitions to subsequent treatments. This may be attributed to perceptions of lower efficacy, leading to them being avoided from the outset unless specific patient needs justify their use.

The full consensus on the future management of therapeutic sequences underscores the need for a strategic, long-term approach to MS treatment [36]. Managing treatment sequences involves balancing clinical and MRI activity control, safety, and tolerability, reflecting the complexity of MS management [22, 37]. Neurologists must reassess and adjust treatment plans over time, considering both short-term and long-term goals as patient needs evolve. Personal considerations, such as family planning [30, 31], should be integrated into sequencing decisions to ensure treatments align with patients’ life goals and do not hinder personal plans.

### Limitations

This study has several limitations. The Delphi methodology, while useful for building consensus, relies on the opinions of a small group of Experts, which may introduce subjective biases. Although the KOLs involved were recognized as experts in the field, the limited sample size reduces the diversity of perspectives and may not fully represent the broader MS community, including patients and healthcare providers. However, the KOLs from the centers participating in this activity follow approximately 50% of Italian patients with MS, ensuring at least partial representativeness of real-world Italian clinical practice, as they also serve as key points of reference for the Italian MS community. Nevertheless, we acknowledge that the inclusion of a more diverse range of participants, including junior clinicians, general neurologists, and patient representatives, could have enriched the breadth and representativeness of the study findings. It should be acknowledged that regional differences within Italy, including variations in reimbursement systems, prescribing restrictions, and infusion center capacity, may influence treatment access and therapeutic decision-making. However, a detailed analysis of these regional healthcare system variables was beyond the scope and objectives of the current study, which primarily aimed to achieve expert consensus on key principles of personalized MS care at the national level, and not on issues specifically related to regional regulatory restrictions or local infusion capacities. Additionally, the study focused on the Italian healthcare landscape, which limits the applicability of the findings to other countries with different healthcare systems, treatment access, and regulations. Accordingly, transferability to other healthcare systems may require adjustments. The consensus was based on a single round of the Delphi process, potentially limiting the depth of discussion and refinement of the statements. Moreover, while the questionnaire was informed by comprehensive data from the *2023 Barometer of Multiple Sclerosis and Related Diseases* [9], the evolving nature of MS therapies means that newer treatments or guidelines not included in the study may impact future therapeutic strategies. For instance, the voting process took place in early 2024, prior to the full clinical integration of some recently approved therapies such as ublituximab. Consequently, these treatments were not considered in the consensus statements and may be addressed in future consensus updates as clinical experience grows.

Additionally, the reliance on a Likert scale for agreement may have oversimplified complex expert opinions on topics like personalized treatment and patient needs. In several instances, responses clustered around the “3” option, indicating moderate agreement but also revealing uncertainty or contrasting views among participants. This pattern was seen in many statements and treatments, pointing to areas that require further discussion or clarification. These limitations should be considered when interpreting the results of the study and their implications for clinical practice.

## Conclusions

Overall, the results of the Delphi voting underscore a strong consensus on the need for a patient-centered approach in MS treatment. Neurologists play a key role in guiding therapeutic decisions, which must be personalized, flexible to accommodate life changes, and involve clear communication with both patients and caregivers. These findings highlight best practices in MS management and provide a roadmap for improving patient outcomes through tailored, effective, and well-communicated therapeutic strategies.

## Data Availability

The corresponding author, who had complete access to all the data of the study, assumes responsibility for the integrity of the data and accuracy in the analysis. The anonymized data set used and analyzed for this study can be obtained from the corresponding author on reasonable request.
